# Oestrogens improve human pancreatic islet transplantation in a mouse model of insulin deficient diabetes

**DOI:** 10.1007/s00125-012-2764-1

**Published:** 2012-11-07

**Authors:** S. Liu, G. Kilic, M. S. Meyers, G. Navarro, Y. Wang, J. Oberholzer, F. Mauvais-Jarvis

**Affiliations:** 1 Department of Medicine, Division of Endocrinology, Metabolism and Molecular Medicine, Northwestern University Feinberg School of Medicine, 303 East Chicago Avenue, Tarry 15-761, Chicago, IL 60611 USA; 2 Present Address: Diabetes Institute, the First Affiliated Hospital of Xiamen University, Xiamen, China; 3 Department of Surgery, Division of Transplant Surgery, University of Illinois at Chicago, Chicago, IL USA; 4 Northwestern Comprehensive Center on Obesity, Northwestern University Feinberg School of Medicine, Chicago, IL USA

**Keywords:** Diabetes, Engraftment, Glucagon, Islets, Oestrogen, Survival, Transplantation

## Abstract

**Aims/hypothesis:**

Pancreatic islet transplantation (PIT) offers a physiological treatment for type 1 diabetes, but the failure of islet engraftment hinders its application. The female hormone 17β-oestradiol (E2) favours islet survival and stimulates angiogenesis, raising the possibility that E2 may enhance islet engraftment following PIT.

**Methods:**

To explore this hypothesis, we used an insulin-deficient model with xenotransplantation of a marginal dose of human islets in nude mice rendered diabetic with streptozotocin. This was followed by 4 weeks of treatment with vehicle, E2, the non-feminising oestrogen 17α-oestradiol (17α-E2), the oestrogen receptor (ER) α agonist propyl-pyrazole-triol (PPT), the ERβ agonist diarylpropionitrile (DPN) or the G protein-coupled oestrogen receptor (GPER) agonist G1.

**Results:**

Treatment with E2, 17α-E2, PPT, DPN or G1 acutely improved blood glucose and eventually promoted islet engraftment, thus reversing diabetes. The effects of E2 were retained in the presence of immunosuppression and persisted after discontinuation of E2 treatment. E2 produced an acute decrease in graft hypoxic damage and suppressed beta cell apoptosis. E2 also acutely suppressed hyperglucagonaemia without altering insulin secretion, leading to normalisation of blood glucose.

**Conclusions/interpretation:**

During PIT, E2 synergistic actions contribute to enhancing human islet-graft survival, revascularisation and functional mass. This study identifies E2 as a short-term treatment to improve PIT.

**Electronic supplementary material:**

The online version of this article (doi:10.1007/s00125-012-2764-1) contains peer-reviewed but unedited supplementary material, which is available to authorised users.

## Introduction

Replacing insulin-producing beta cells by human pancreatic islet transplantation (PIT) offers a physiological therapeutic approach for type 1 diabetes. PIT benefits recipients in a way that insulin therapy can never achieve [1–3]. Currently, most immunosuppressed type 1 diabetic recipients of human islets allotransplant [3, 4] can successfully attain insulin independence and protection from hypoglycaemia at 1 year. However, there are critical limitations to the widespread application of PIT. First, procuring sufficient islet yield requires several deceased human donors and novel strategies are needed to achieve insulin independence with fewer islets [4]. Evidence suggests that as little as 20% of the islets within a pancreas are required to maintain euglycaemia [5]. Still, the islets of one or two pancreases are needed to reverse diabetes following PIT, suggesting that a significant portion of the transplanted islets are destroyed or become non-functional. This early islet loss is believed to represent up to 70% of the transplanted islet mass [6–8]. The transplanted islets are avascular, and islet revascularisation takes 2–3 weeks to complete. During that period, the islets are vulnerable to hypoxic stress and may be destroyed before revascularisation and engraftment occur [9, 10]. Thus, in the immediate post-transplant period, apoptosis and failure of vascular engraftment pose a challenging problem. We must explore new therapeutic approaches promoting islet survival and revascularisation to enhance islet engraftment.

Recently, the female hormone, 17β-oestradiol (E2) has been shown to protect mouse and human islets from apoptosis induced by multiple injuries and to stimulate insulin biosynthesis via oestrogen receptors (ERs) present in beta cells [11–18]. E2 also stimulates endometrial angiogenesis and promotes endothelial cell recovery after injury [19–23]. Together, these findings raise the prospect that E2 may be applied to improve islet functional mass and revascularisation during PIT. Here, we used an insulin-deficient mouse model with xenotransplantation of a marginal dose of human islets to test the hypothesis that oestrogens improve islet engraftment during PIT.

## Methods

### Animals and the induction of experimental diabetes

Diabetes was induced in 10-week-old male nude mice (C57BL/6; Harlan Laboratories, Indianapolis, IN, USA) or male mice null for *Erα* [24] by a single i.p. injection of 200 mg/kg streptozotocin (STZ; Sigma Aldrich, St Louis, MO, USA). Blood glucose was monitored with a One Touch Ultra Glucose Monitor (LifeScan, Milpitas, CA, USA). Mice with fed blood glucose exceeding 20.8 mmol/l were used as recipients. All animal experiments were approved by Northwestern University Animal Care and Use Committee.

### Human islet transplantation and in vivo oestrogen treatment

Human islets were obtained through the Integrated Islet Distribution Program and cultured for 48 h in phenol-red-free CMRL-1066 medium (Sigma Aldrich) containing 10% (vol./vol.) charcoal-stripped FBS (Gemini Bio, West Sacramento, CA, USA). For in vivo treatment, E2, 17α-oestradiol (17α-E2) and G1 (0.18 mg/pellet), propyl-pyrazole-triol (PPT) and diarylpropionitrile (DPN) (3.6 mg/pellet) 60-day-release pellets (Innovative Research of America, Sarasota, FL, USA) were implanted subcutaneously immediately before islet transplantation. The dose of E2 was chosen to obtain serum concentrations within physiological limits (ranging from oestrus to pregnancy). The doses of 17α-E2 and G1, PPT and DPN were chosen according to the affinity of each agonist for its ER to achieve similar activation of ERs as caused by E2. A marginal dose of 1,000 islet equivalents (IEQ) of human islets were transplanted under the kidney capsule of recipient mice.

### Immunosuppression

Immunosuppression was achieved as described in the Edmonton protocol, following adaptation to the mouse [1, 25]. Sirolimus (rapamycin; LC Laboratories, Woburn, MA, USA) was administered via i.p. injection every other day at the dose of 0.1 mg/kg for 4 weeks, starting from the day of islet transplantation. Tacrolimus (FK506; Cayman, Ann Arbor, MI, USA) was administered i.p. daily at 1 mg/kg, starting on the day of islet transplantation. Control animals received daily vehicle injections.

### Immunohistochemistry

Kidneys bearing islet grafts were fixed overnight in 4% (wt/vol) paraformaldehyde at 4°C. The tissues were immersed in 30% (wt/vol) sucrose and embedded in tissue-freezing medium (Tissue-Tek; Sakura Finetek, Torrance, CA, USA). Sections, 5–10 μm, were mounted on charged slides. For immunohistochemical studies, the following primary antibodies were used: guinea pig anti-human insulin (1:1,000; Linco Research, Saint Charles, MO, USA); rat anti-mouse CD31 (1:400; BD Biosciences, San Jose, CA, USA); rabbit anti-mouse Ki67 (1:400; Novocastra, Newcastle Upon Tyne, UK); rat anti-mouse F4/80 (1:200; AbD Serotec, Raleigh, NC, USA); mouse anti-ERα (1D5; 1:100; Zymed Laboratories, South San Francisco, CA, USA); and goat anti-ERβ (Y-19; 1:100; Santa Cruz Biotechnology, Santa Cruz, CA, USA). The secondary antibodies were: Cy3-conjugated donkey anti-guinea pig; Cy3-conjugated goat anti-rat; biotinylated goat anti-rat; FITC-conjugated goat anti-rat; and FITC-conjugated donkey anti-rabbit (Jackson ImmunoResearch Laboratories, West Grove, PA, USA). Biotinylated goat anti-rat was visualised using the Vectastain Elite ABC kit (Vector Laboratories, Burlingame, CA, USA). For staining with ERα and ERβ, the Alexa 568 tyramide signal amplification kit (Molecular Probes, Eugene, OR, USA) was used. For nuclear staining, the sections were incubated with DAPI (Molecular Probes, Eugene, OR, USA). Images were obtained with a Nikon Eclipse E400 microscope (Nikon Instruments, Melville, New York, USA) or TissueGnostics High Throughput Imaging System (TissueGnostics, Vienna, Austria).

### Measurement of apoptosis using TUNEL assay

Apoptotic cells were detected by TUNEL assay using a fluorescein in situ cell death detection kit (Roche, Indianapolis, IN, USA) 1 day after transplantation. Frozen tissue sections, 5 μm, were fixed at room temperature for 1 h in 4% (vol./vol.) PFA in PBS, pH 7.4. Samples were washed in PBS for 30 min. Following the manufacturer’s instructions, sections were permeabilised, washed, labelled, incubated and analysed. Sections were subsequently stained with guinea pig anti-human insulin as a primary antibody (1:1,000) and Cy3-conjugated donkey anti-guinea pig as a secondary antibody. Images were obtained with a Nikon Eclipse E400 microscope.

### Measurement of oxygenation in transplanted islet graft

Islet oxygenation was investigated in the transplants, 1 day post-transplantation [26]. Pimonidazole hydrochloride (hpi Hydroxyprobe-1; Omni Kit, Burlington, MA, USA) (60 mg/kg) was injected i.p. Mice were killed 3 h later, and their kidney containing the islet graft was processed for immunohistochemistry. Rabbit anti-pimonidazole antibody (hpi Hydroxyprobe-1; Omni Kit) was used to visualise pimonidazole hydrochloride accumulation in hypoxic cells of the graft.

### Morphometric analysis

Kidneys bearing islet grafts were sectioned in 10 μm thickness and four sections per tissue were randomly chosen for morphometric analysis. Anti-human-insulin and anti-mouse-CD31 antibodies were used to visualise beta cells and blood vessels, respectively. Morphometric analysis was conducted using the ImageJ 1.37v (rsb.info.nih.gov/ij/) program. In islet transplants, the beta cell area was calculated by dividing the insulin-positive area by the graft area. Blood vessel density was calculated by dividing the mouse-CD31-positive area by the graft area [27]. The demarcation of an islet graft was considered to be the parenchyma of the surrounding kidney as described [10].

### Hormone and thiobarbituric acid reactive substances assays

Human insulin and mouse glucagon was measured in serum by ELISA and RIA, respectively (Linco, St Charles, MO, USA). Systemic oxidative stress was assessed by measuring the concentration of thiobarbituric acid reactive substances (TBARS) in plasma (Cayman) and kidney homogenates (ZeptoMetrix, Franklin, MA, USA) [28].

### Lectin infusion

Biotinylated tomato lectin (Vector Laboratories) was injected into the tail vein (200 μg) and allowed to circulate for 5 min before the mice were killed. The kidneys bearing the islet grafts were removed and prepared for histological study. Lectin staining was visualised using the Vectastain Elite ABC kit (Vector Laboratories). Functional blood vessel density was obtained by dividing the lectin-positive area by the graft area.

### Statistical analysis

Data are presented as mean ± SEM unless otherwise stated. For the rodent study, data were analysed by either the unpaired Student’s *t* test or one-way ANOVA. A value of *p* < 0.05 was considered statistically significant.

## Results

### Oestrogens ameliorate diabetes

**Fig. 1:**
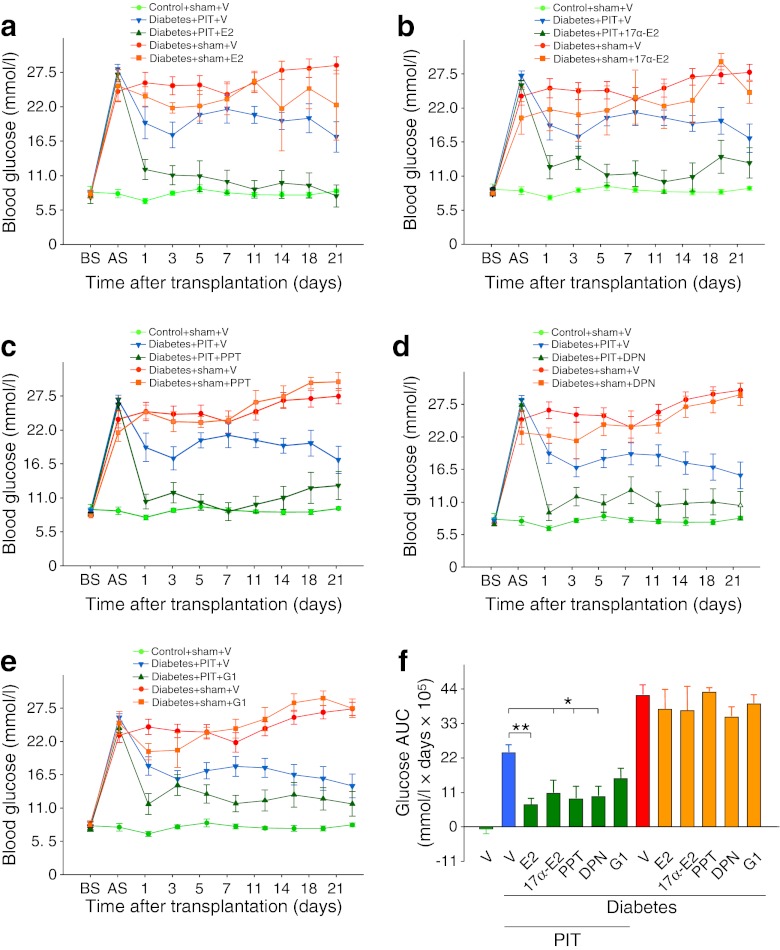
Oestrogens ameliorate diabetes after PIT. Effect of (**a**) E2, (**b**) 17α-E2, (**c**) PPT, (**d**) DPN and (**e**) G1 on blood glucose after PIT. (**f**) Blood glucose AUC was calculated from (**a–e**). Values represent the mean ± SEM, *n* = 4–18/group. ^*^ *p* < 0.05, ^**^ *p* < 0.01. AS, after STZ; BS, before STZ; V, vehicle

We investigated the therapeutic action of oestrogens on islet transplantation outcomes. We used male immunodeficient nude mice rendered totally insulin deficient by injection of a single high dose of STZ (200 mg/kg). In these mice, we performed either sham transplantation or xenotransplantation of a marginal dose of human islets (1,000 IEQ) under the renal capsule (PIT). This was followed by treatment with vehicle or E2, leading to serum concentrations within the physiological range (E2 levels: vehicle 68.49 ± 25.5 pg/ml; E2 injection 321.93 ± 77.15 pg/ml), the non-feminising E2 stereoisomer 17α-E2 [29], the ERα-selective agonist PPT [30], the ERβ-selective agonist DPN [31] and the G protein-coupled oestrogen receptor (GPER) agonist G1 [32]. Following PIT or sham transplantation, blood glucose was monitored for 3 weeks. PIT mice treated with vehicle remained hyperglycaemic, but conversely, PIT mice treated with E2, 17α-E2, PPT, DPN or G1 showed a dramatic improvement in blood glucose (Fig. 1a–f) starting 1 day after transplantation. In sham-operated diabetic mice, E2, 17α-E2, PPT, DPN and G1 treatment had no effect on blood glucose (Fig. 1a–f) or body weight (electronic supplementary material [ESM] Fig. 1) compared with vehicle. This finding demonstrated that the improvement in blood glucose by ER agonists required the presence of transplanted human islets. A similar protection of human PIT was observed in female nude mice treated with E2 (ESM Fig. 2), demonstrating that E2 protection is not sex specific. Thus, we performed the rest of the study in male mice.

### Oestrogens enhance islet engraftment

**Fig. 2:**
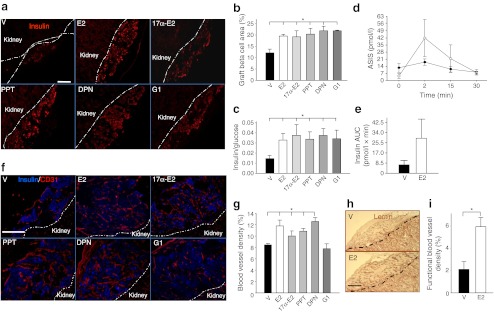
Oestrogens improve islet engraftment after PIT. (**a**) Representative sections showing immunofluorescence staining for insulin-positive cells in islet grafts. (**b**) Quantification of graft beta cell area (insulin-positive area/total graft area). (**c**) Ratio of random-fed insulin (pmol/l)/glucose (mmol/l) was used as an index of insulin production. (**d**) Insulin response to l-arginine (ASIS) (black, vehicle; white, E2) following i.p. l-arginine injection (3 g/kg). (**e**) AUC for ASIS from (**d**). (**f**) Representative sections of mouse CD31 immunofluorescence staining in islet grafts. (**g**) Quantification of graft blood vessel density (CD31-positive area/graft area). (**h**) Representative sections showing lectin staining in islet grafts. (**i**) Quantification of graft functional blood vessel density (lectin-positive area/graft area). In (**a**) and (**f**) the cell nuclei were stained with DAPI (blue). Values represent the mean ± SEM, *n* = 3–9/group. ^*^ *p* < 0.05. Scale bars, 100 μm. V, vehicle

At 4 weeks after PIT, mice treated with E2, 17α-E2, PPT, DPN or G1 showed protection of islet-graft beta cell mass (Fig. 2a, b) associated with a rise in human serum insulin concentrations compared with vehicle-treated PIT mice (Fig. 2c). Insulin response to i.p. arginine was also increased in the E2- compared with the vehicle-treated mice (Fig. 2d, e). Additionally, the endogenous pancreatic beta cell mass of STZ-treated PIT recipient mice was efficiently reduced and unaffected by ER agonist treatments (ESM Fig. 3). Thus, activation of ERα, ERβ and GPER with oestrogens preserves functional islet mass and reverses diabetes after PIT.

We next quantified the endothelial cell area in islet grafts using the mouse endothelial cell marker CD31. At 4 weeks after PIT, mice treated with E2, 17α-E2, PPT or DPN showed an improvement in islet revascularisation compared with vehicle-treated mice (Fig. 2f, g). Conversely, the mice treated with G1 did not show any improvement compared with controls (Fig. 2f, g). To determine the functionality of the E2-induced reconstructed islet vasculature network, we applied in vivo staining for lectin—a commonly used marker of vessel functionality [33]—to a group of mice treated with vehicle or E2. We detected increased lectin staining in E2-treated mice compared with vehicle-treated mice (Fig. 2h, i), demonstrating that E2 increased functional vessel density in the transplanted islets. While the contribution of endothelial cells from freshly isolated donor islets to the formation of functional vessels within islet grafts has been demonstrated [33, 34], cultured islets lose most of their endothelial cell populations within 3 days of culture [34]. We thus examined whether E2 treatment could preserve the intra-islet endothelial cell population in freshly isolated mouse islets. Consistent with a previous report [34], we observed that the islet endothelial cell number decreased within 1 day of culture and totally disappeared after 3 days. However, we observed no beneficial effect of E2 treatment on the maintenance of intra-islet endothelial cell population (ESM Fig. 4). As E2 favours islet survival, we tested whether in vitro E2 treatment of human islets prior to PIT could improve PIT outcome using the same model system. We observed that E2 treatment of cultured human islets prior to PIT had no significant effect on subsequent glycaemic control either alone or in association with in vivo E2 treatment (ESM Fig. 5).

### Improvement of islet engraftment persists in the presence of immunosuppression and after discontinuation of E2 treatment

**Fig. 3:**
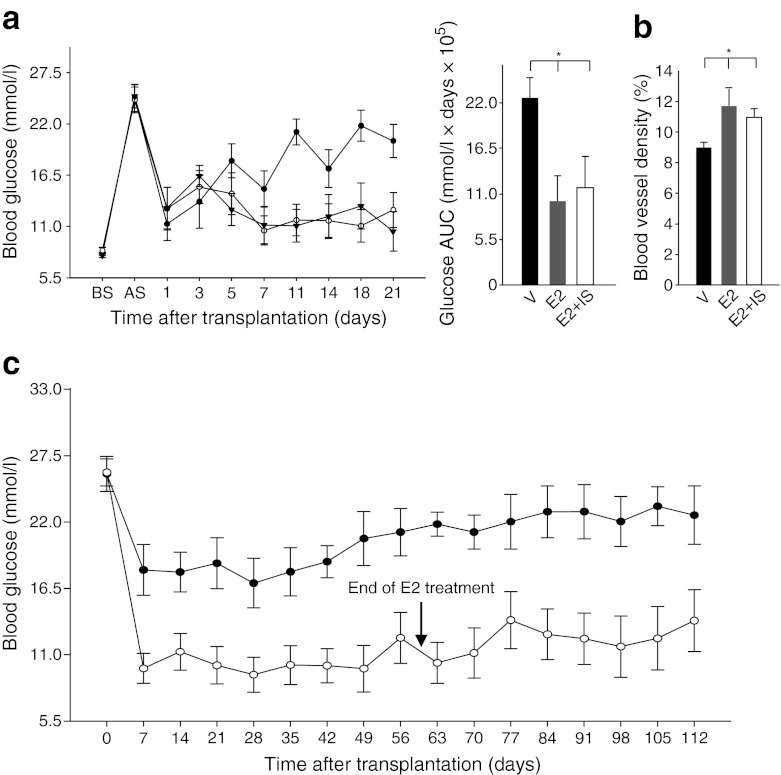
Improvement of PIT persists in the presence of immunosuppression and after discontinuation of E2 treatment. (**a**) Line graph showing the effect of E2 on blood glucose in the presence of immunosuppressive treatment (black circles, PIT+V; white circles, PIT+E2; black triangles, PIT+E2+ immunosuppressant), and bar chart showing blood glucose AUC as calculated from the graph. (**b**) Quantification of graft blood vessel density (CD31-positive area/graft area) in mice from (**a**). (**c**) Blood glucose was measured in vehicle-treated (black circles) and E2-treated (white circles) mice on the indicated days. Values represent the mean ± SEM, *n* = 7–9/group. ^*^ *p* < 0.05. AS, after STZ; BS, before STZ; IS, immunosuppressant; V, vehicle

The Edmonton protocol has facilitated human islet transplantation by using non-steroidal immunosuppressive treatment such as sirolimus (rapamycin) and tacrolimus (FK-506) [1, 3]. However, these regimens undermine angiogenesis and represent a significant obstacle to islet engraftment [35, 36]. To determine the persistence of E2 improvement of islet engraftment after PIT in the presence of immunosuppression, we treated a group of mice with sirolimus and tacrolimus for 4 weeks in the presence of vehicle or E2. The treatments did not alter E2-induced improvement of glycaemic control and islet revascularisation (Fig. 3a, b), demonstrating that E2 improves PIT in the presence of immunosuppression. As chronic E2 treatment following islet transplantation is not suitable in the clinical setting, we sought to determine whether glycaemic control could be sustained after E2 discontinuation. We followed a group of PIT mice for 112 days, during which time we maintained the E2 treatment for the first 60 days. Our results show that E2 efficacy on PIT outcome, assessed by normalisation of blood glucose, lasted for 50 days after E2 discontinuation (Fig. 3c). These findings suggest that transient E2 treatment has induced a permanent improvement in functional islet mass and engraftment.

### E2 acutely improves islet-graft hypoxia

**Fig. 4:**
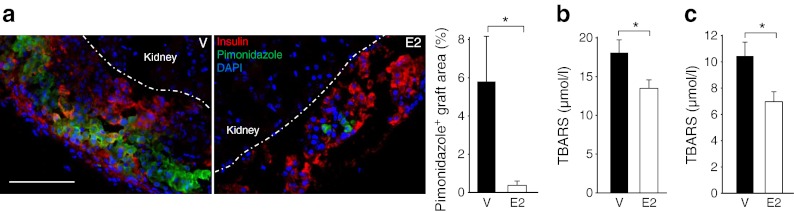
E2 acutely improves islet-graft hypoxia. (**a**) Representative pictures of pimonidazole staining (green) in the islet grafts, 1 day after PIT, and bar chart showing pimonidazole-positive area in the graft. In the images, red fluorescence has been used for insulin. The cell nuclei were stained with DAPI (blue). Lipid peroxidation was determined in (**b**) plasma and in (**c**) islet-bearing kidney homogenates, 1 day after PIT, by TBARS assay. Values represent the mean ± SEM, *n* = 3–8/group. ^*^ *p* < 0.05. Scale bars, 50 μm. V, vehicle

The acute correction of hyperglycaemia observed 1 day after transplantation (Fig. 1)—a time when revascularisation has not yet taken place—suggested that oestrogens had acutely improved islet survival and/or function independently from the graft revascularisation. Because the process of islet transplantation elicits an acute inflammatory response, we first looked at whether E2 had acutely suppressed graft inflammation. We observed no difference between the E2- and vehicle-treated groups in graft macrophage infiltration (ESM Fig. 6). E2 improves splanchnic macro- and microcirculation during haemorrhagic shock [37]. A similar improvement in renal cortex microcirculation would improve islet-graft oxygenation, thus improving survival and function at this early stage when islets are not revascularised. To explore this possibility, we quantified graft hypoxia using pimonidazole [26] at 1 day after PIT. We observed a marked decrease in the hypoxic area in E2-treated grafts (Fig. 4a). Hyperglycaemia and hypoxia provoke the formation of oxygen free radicals and lipid peroxidation that trigger islet apoptosis. To measure oxidative stress, we quantified lipid peroxidation in serum and kidney tissue adjacent to the graft using the TBARS method [28]. We observed decreased serum and kidney TBARS concentration in E2-treated mice, demonstrating that E2 treatment has improved hypoxic stress in these mice (Fig. 4b, c).

### E2 acutely suppresses graft beta cell apoptosis

**Fig. 5:**
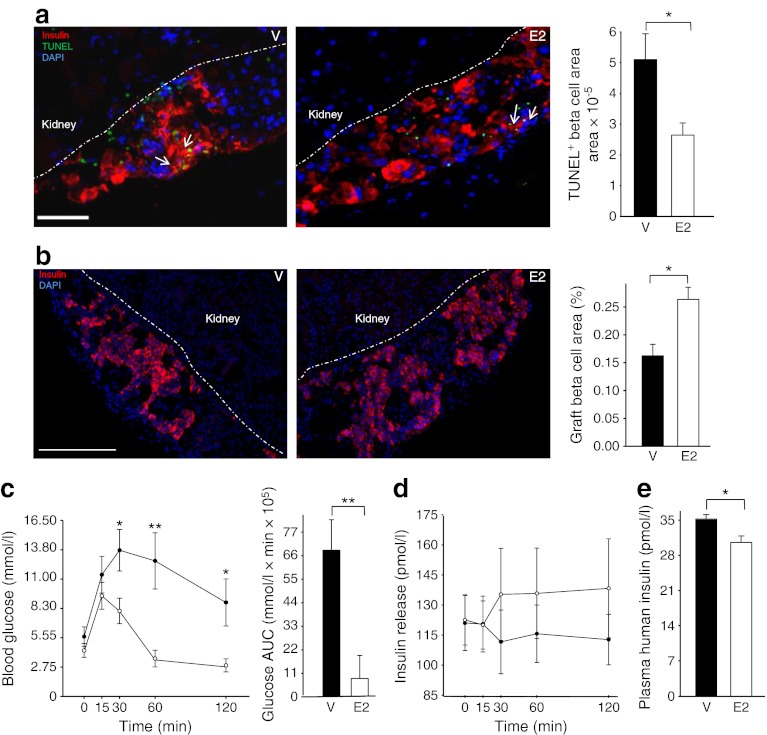
E2 treatment acutely prevents beta cell apoptosis. (**a**) Representative sections showing immunofluorescence staining for TUNEL-positive (green) and insulin-positive (red) cells in islet grafts, 1 day after PIT. Arrows show TUNEL-positive beta cells. Bar charts show quantification of TUNEL-positive/beta cells (insulin-positive) area in the graft. Scale bar, 25 μm. (**b**) Representative sections showing immunofluorescence staining for insulin-positive (red) cells in islet grafts. Bar charts showing the percentage of insulin-positive area in graft. Scale bar 100 μm. (**c**) Line graph showing results of i.p. glucose tolerance test (2 mg/kg body weight; black circles, vehicle; white circles, E2), and bar chart with corresponding AUC for glucose. (**d**) Insulin secretion after i.p. GTT (black circles, vehicle; white circles, E2). (**e**) Plasma human insulin levels. In (**a**) and (**b**) the cell nuclei were stained with DAPI (blue). In (**b**), (**c**), (**d**) and (**e**) measurements were made on day 3 after PIT. Values represent the mean ± SEM, *n* = 5–11/group. ^*^ *p* < 0.05, ^**^ *p* < 0.01. V, vehicle

We next studied graft beta cell turnover and function in the early post-transplant period. We observed that E2 treatment acutely suppressed apoptosis (day 1 after PIT; Fig. 5a), without an effect on beta cell proliferation (ESM Fig. 7). This was associated with a greater beta cell graft area (day 3 after PIT; Fig. 5b), suggesting a retained beta cell mass. E2 treatment dramatically improved i.p. glucose tolerance in PIT mice (Fig. 5c) but, surprisingly, despite the retained beta cell area, E2 acute suppression of blood glucose was not associated with increased glucose-stimulated insulin secretion (Fig. 5d) and random-fed insulin concentrations were reduced (Fig. 5e).

### PPT acutely suppresses blood glucose in the absence of ERα in the islet graft

**Fig. 6:**
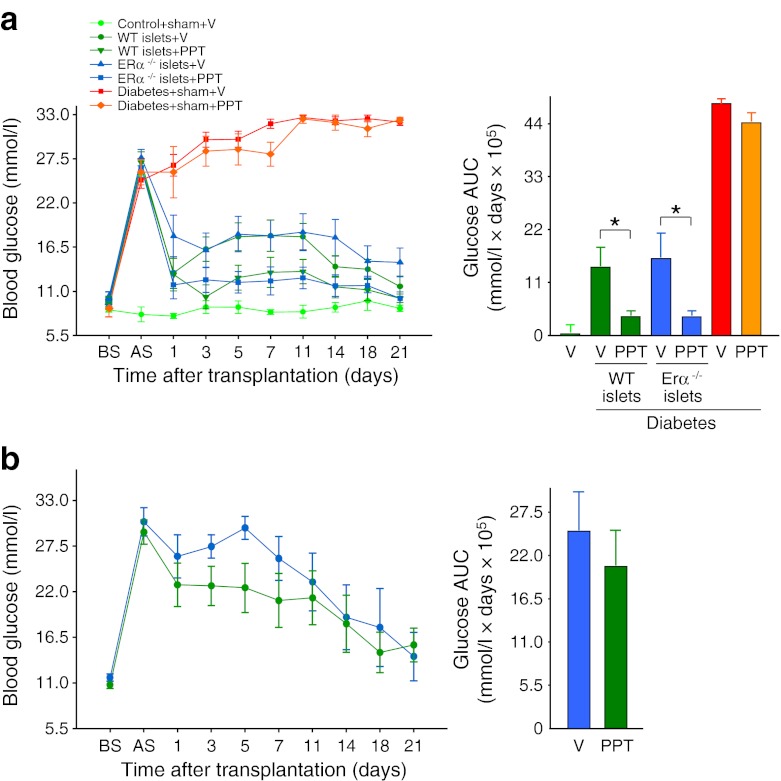
E2 acutely suppresses blood glucose via ERα activation outside the islet graft. (**a**) Line graph showing the effect of PPT on blood glucose after transplantation of WT and *Erα* ^−/−^ mouse islets in WT recipient mice and bar chart showing corresponding glucose AUC. (**b**) Line graph showing the effect of PPT on blood glucose after transplantation of WT mouse islet in *Erα* ^−/−^ recipient mice (blue, vehicle; green, PPT), and bar chart showing the corresponding blood glucose AUC. Values represent the mean ± SEM, *n* = 3–14/group. ^*^ *p* < 0.05. AS, after STZ; BS, before STZ; V, vehicle

As E2 suppression of blood glucose was independent from an enhanced insulin secretion from the islet graft, we asked whether this effect required direct activation of ER in the islet graft or ER activation in the recipient tissue (leading to increased insulin sensitivity). To address this issue, we used ERα as a paradigm of E2 actions and mice with global null deletion of *Erα* (*Erα* ^−/−^ mice). We first performed an allotransplantation of a marginal dose of islets (150) isolated from donor littermate wild-type (WT) or *Erα* ^−/−^ mice under the kidney capsule of recipient WT mice. These recipient mice were treated with vehicle or the ERα-selective agonist PPT. As expected, WT mice transplanted with WT or *Erα* ^−/−^ islets and treated with vehicle remained hyperglycaemic. However, PPT treatment led to a similar early decrease in blood glucose in WT mice transplanted with WT or *Erα* ^−/−^ islets, demonstrating that PPT improves PIT in the absence of islet ERα (Fig. 6a). Next, we performed an allotransplantation of a marginal dose of WT islets in recipient *Erα* ^−/−^ mice followed by treatment with vehicle or PPT. Vehicle- and PPT-treated mice showed a similar and minor blood-glucose-lowering effect due to the transplantation of WT islets in *Erα* ^−/−^ mice. However, PPT treatment did not produce a stronger hypoglycaemic effect than vehicle in these ERα-deficient mice. Together, these data demonstrate the requirement of ERα activation in the recipient mouse tissues to acutely suppress blood glucose (Fig. 6b).

### E2 acutely suppresses glucagon production

**Fig. 7:**
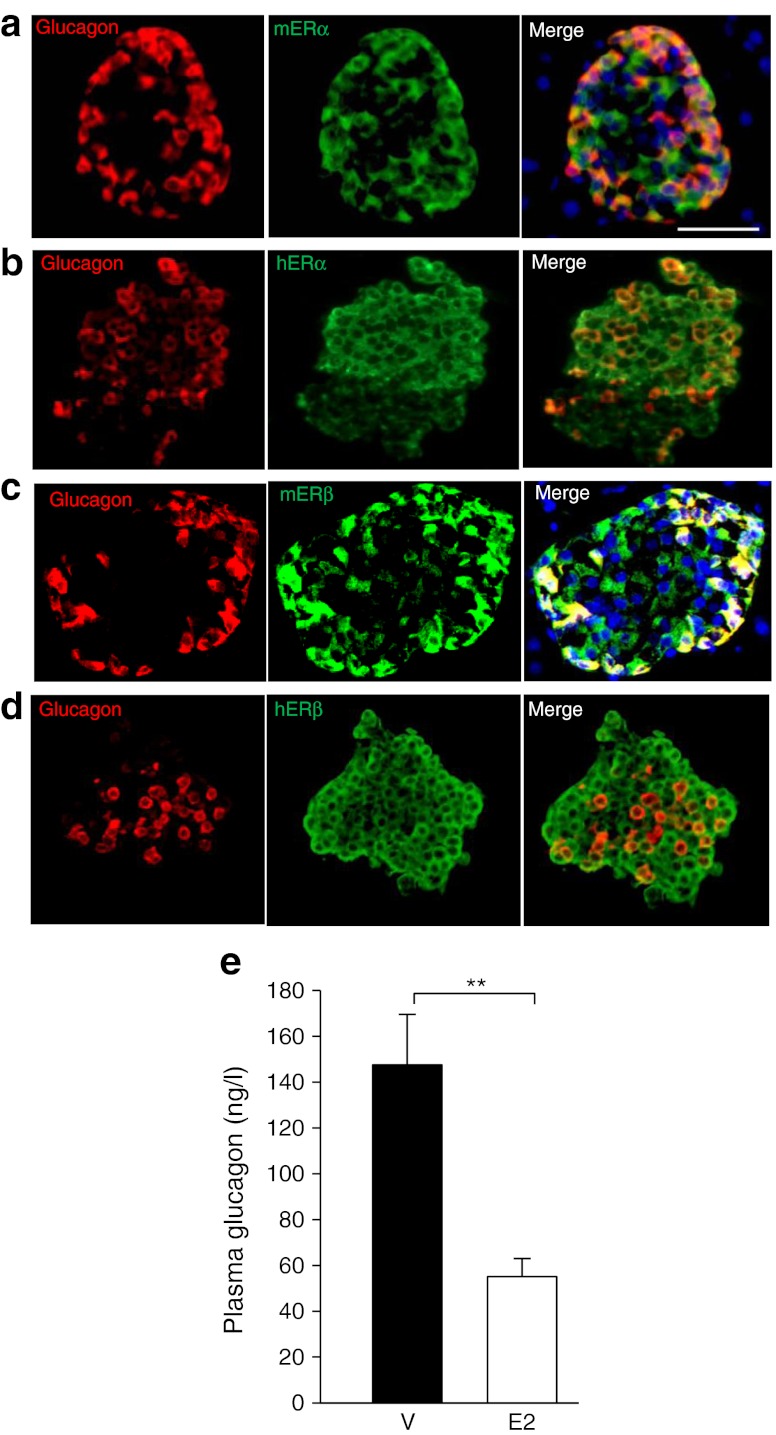
E2 acutely suppresses hyperglucagonaemia. Representative pictures of islets showing co-localisation of (**a**) mouse ERα, (**b**) human ERα, (**c**) mouse ERβ, and (**d**) human ERβ (green) with alpha cells stained with glucagon (red). In (**a**) and (**c**), the cell nuclei were stained with DAPI (blue). (**e**) Plasma glucagon levels at day 1 after PIT. Values represent the mean ± SEM, *n* = 3–5/group. ^**^ *p* < 0.01. Scale bar, 25 μm. h, human; m, mouse; V, vehicle

That treatment with the ERα agonist PPT produced an acute decrease in blood glucose in the absence of ERα in the islet graft and without an increase in insulin secretion suggests that PPT acts on the host alpha cells to suppress glucagon secretion. Indeed, unsuppressed hyperglucagonaemia plays a major role in hyperglycaemia in all forms of insulin-deficient diabetes [38, 39]. To address this issue, we looked at the effect of E2 treatment on serum glucagon in the early transplant period. First, we observed that ERα and ERβ were present in alpha cells from mice and human islets (Fig. 7a–d). In glucagon-secreting INR1G9 cells and αTC6 cells, the 67 kd-long isoform of ERα was produced at a level similar to that in insulin-secreting MIN-6 cells (ESM Fig. 8). In addition, we observed that 1 day after PIT, E2 treatment was associated with a dramatic suppression of serum glucagon compared with vehicle-treated mice in random-fed state (−65%, Fig. 7e), thus providing an explanation for the simultaneous decrease in glucose (Fig. 1a) and insulin levels (Fig. 5e) associated with E2 treatment.

## Discussion

The present study shows that E2, used at physiological concentrations, improves islet engraftment in a sex-non-specific manner in an insulin-deficient nude mouse model transplanted with a marginal dose of human islets and in the absence of allorejection or autoimmunity recurrence. Several non-exclusive mechanisms could be implicated.

### Suppression of hyperglucagonaemia

The acute correction of hyperglycaemia and improvement of glucose tolerance observed 1 day after transplantation and following E2 treatment occur without an increase in graft insulin secretion. In fact, E2 treatment produces a decrease in fed serum insulin concentrations, suggesting that E2 has improved insulin sensitivity. Indeed, the E2 hypoglycaemic action occurs in the face of better suppression of serum hyperglucagonaemia. This observation suggests that the E2-mediated acute suppression of blood glucose is independent of an improvement in beta cell function; rather, it is dependent on glucagon suppression. This may explain why E2-treated mice have suppressed hyperglucagonaemia and lower insulin concentrations, as a decrease in hyperglucagonaemia improves glucagon-induced hepatic insulin resistance. This observation is also consistent with the ‘glucagonocentric’ vision of diabetes pathophysiology in which glucagon excess, rather than insulin deficiency, is the sine qua non of hyperglycaemia in all forms of diabetes, at least in rodents [40]. Indeed, total insulin deficiency by beta cell destruction in glucagon-receptor-null mice, which are unresponsive to glucagon, does not cause diabetic abnormalities [41]. We observe that ERα and ERβ are produced in mouse and human alpha cells, further indicating that alpha cells are direct targets of E2 actions. In addition, our experiments suggest that ERα needs to be activated in alpha cells of the host pancreas as the hypoglycaemic effect of an ERα-selective agonist is lost when islets from WT mice are transplanted in Erα null mice (lacking ERα in alpha cells). Accordingly, the ERα agonist can still acutely suppress blood glucose and permanently improve diabetes when ERα-deficient islets are transplanted in WT mice. This demonstrates that ERα action in graft alpha cells is not necessary to suppress blood glucose. Recently, leptin has been shown to suppress glucagon and correct diabetes in mice, in the absence of insulin [42]. Our results suggest that E2 requires the presence of insulin—or at least functional beta cells—to suppress glucagon as ER agonists have no hypoglycaemic effect in diabetic mice in the absence of transplanted islets. This observation is consistent with the concept that insulin suppresses glucagon secretion from alpha cells via paracrine mechanisms [43] and that insulin signalling in alpha cells is required for this process [44]. Thus, in the alpha cells of diabetic mice E2 may act as an insulin sensitiser in suppressing glucagon production. Additional studies in mice lacking ERs in alpha cells are needed to address this issue.

### Protection of functional beta cell mass

Oestrogens have acutely improved islet survival following transplantation, as demonstrated by E2-mediated acute suppression of graft apoptotic beta cells. This is the first evidence that E2, used at therapeutic doses, protects human islets from apoptosis in an in vivo diabetic environment. We and others have shown that E2 promotes islet survival in conditions of oxidative stress and pro-inflammatory cytokine injury in culture and in vivo [11–13, 17]. Higher islet functionality and decreased apoptosis are also observed after transplantation of rat islets recovered from E2-treated brain-dead donors [45]. This anti-apoptotic protection probably accounts for the early retention of graft beta cell mass, which persists after 3 weeks of ER agonist treatment. In addition, we believe that E2 anti-apoptotic protection is also mediated via activation of ERs in recipient endothelial cells. E2 dilates the mesenteric arteries and increases mesenteric blood flow [46, 47]. E2 also improves splanchnic circulation during haemorrhagic shock, which increases oxygenation [37]. We observed that E2 acutely and dramatically improved islet-graft hypoxia, which is associated with decreased systemic and kidney oxidative damage. This protection of islet oxygenation is probably instrumental in E2 acute anti-apoptotic protection as, following PIT, hypoxic stress is a critical factor in the early loss of islet mass [8–10]. Initially, E2-treated graft beta cells are non-functional and show no insulin-secretory response to the glucose challenge, probably as a result of hyperglycaemia-induced glucose desensitisation and/or lack of islet vascularisation. However, following 3 weeks of normoglycaemia, PIT mice treated with ER agonists show a restoration of beta cell function. Thus, E2 acute normalisation of blood glucose, via suppression of hyperglucagonaemia, has favoured the subsequent restoration of beta cell function following correction of glucose desensitisation.

### Action on recipient endothelial cells

Apart from E2-mediated acute improvement of islet-graft oxygenation, the likely action of oestrogens involves the enhancement of islet revascularisation through action on recipient endothelial cells, as E2 is a potent angiogenic factor in the endometrium and following vascular injury [22, 23]. Accordingly, E2 increases the functional blood vessel density of the human islet graft. More importantly, immunosuppression with sirolimus and tacrolimus typically inhibits islet revascularisation and engraftment. Yet E2 stimulation of revascularisation persists in the presence of this immunosuppression regimen. Furthermore, after discontinuation of E2 treatment the correction of diabetes endures, demonstrating that E2 has induced a stable engraftment. E2 treatment promotes revascularisation of the transplanted human islets through ERs on the endothelial cell from the recipient kidney cortex and without the participation of endothelial cells from the human graft, as these cells had disappeared at the time of transplantation. However, G1, which exhibits potent anti-apoptotic action in human islets [13], improves PIT outcome without enhancing graft revascularisation, suggesting that the acute hypoglycaemic and anti-apoptotic effects of oestrogens are more important to the engraftment process than the moderate increase in revascularisation.

**Fig. 8:**
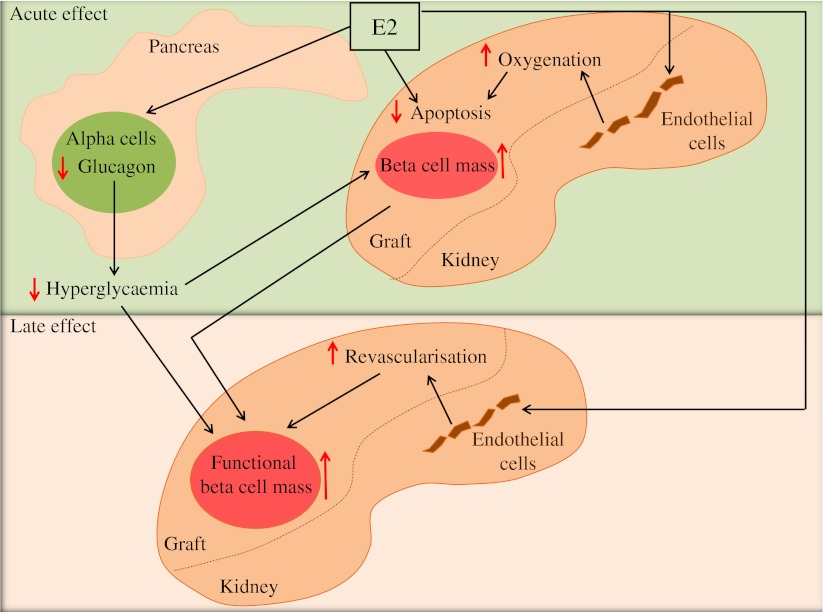
Schematic representation of the proposed mechanisms of E2 improvement of islet engraftment. E2 exhibits acute actions in recipient endothelial cells, decreasing islet-graft hypoxic stress, that synergise with E2 acute action in graft beta cells to prevent apoptosis. In addition, E2 exhibits acute actions in recipient alpha cells suppressing hyperglucagonaemia and hyperglycaemia. These acute actions help retain the initial beta cell mass. E2 also exhibits late actions on graft endothelial cells to improve revascularisation. These acute and late E2 actions concur to promote islet engraftment and maintenance of functional beta cell mass

Thus, the synergistic actions of E2 on ERs in recipient alpha cells, endothelial cells and graft beta cells leading, respectively, to the suppression of hyperglucagonaemia and hyperglycaemia, the protection from islet-graft hypoxia, oxidative stress and apoptosis and ultimately to revascularisation of the graft, have concurred to promote islet engraftment and maintenance of beta cell functional mass. A summary of the proposed mechanisms of E2 action is shown in Fig. 8.

Our findings have direct therapeutic implications for PIT in type 1 diabetes. Although long-term E2 treatment following PIT is not suitable because of the risk of cancer, conversely, transient E2 treatment represents a safe and immediately available alternative to improve PIT in women. Indeed, fertile women with type 1 diabetes show E2 deficiency compared with healthy women [48]. Therefore, women with type 1 diabetes undergoing islet transplantation have lost their endogenous E2 protection and could benefit from short-term oestrogen supplementation using therapeutic doses. Furthermore, 17α-E2, which is endogenous in humans, has few of the biological effects associated with the female hormone activity and may be a candidate for sex-neutral therapy in PIT.

In conclusion, E2 enhances islet engraftment after PIT. As oestrogens are approved by the Food and Drug Administration, further testing in women with the addition of oestrogens to the Edmonton protocol should be considered. This could provide an immediate therapeutic alternative to improve PIT and achieve insulin independence with fewer islets, long before other surrogate islet beta cell sources or beta cell regeneration therapy can be developed.

## Electronic supplementary material

Below is the link to the electronic supplementary material.

ESM Figure 1 Body weight after PIT. Effect of (a) E2, (b) 17α-E2, (c) PPT, (d) DPN and (e) G1 on body weight after PIT. Values represent the mean ± SEM. *n* = 4–18/group. AS, after STZ; BS, before streptozotocin; V, vehicle. (PDF 131 kb)

ESM Figure 2 Oestrogens ameliorate diabetes in female mice after PIT. (a) Effect of oestrogen on blood glucose levels in female mice (black circles, vehicle; white circles, E2). (b) Blood glucose area under the curve (AUC) was calculated from (a). Values represent the mean ± SEM, *n* = 4/group. *p < 0.05, **p < 0.01. AS, after STZ; BS, before streptozotocin; V, vehicle. (PDF 12 kb)

ESM Figure 3 Effect of oestrogens on pancreatic beta cell mass. Pancreatic beta cell mass was measured in sham operated control mice and PIT recipient mice treated with E2, 17α-E2, PPT, DPN, and G1. Values represent the mean ± SEM, *n* = 4–6/group. *** *p* < 0.001. V, vehicle. (PDF 70 kb)

ESM Figure 4 Effect of in vitro E2 treatment on the retention of intra-islet endothelial cell population. (a) Representative sections showing immunofluorescence staining for mouse CD31+ (red) cells in cultured mouse islets (green). (b) Quantification of endothelial cell population in islets. Values represent the mean ± SEM, *n* = 3/group. V, vehicle. (PDF 50 kb)

ESM Figure 5 Effect of in vitro E2 treatment on the PIT outcome. (a) Effect of in vitro E2 treatment alone (10^−8^ M) on blood glucose after PIT (blue circle, vehicle; green circle, E2). (**b**) Blood glucose area under the curve (AUC) from (a). (c) Comparison of dual in vitro and in vivo E2 treatment with in vivo E2 treatment alone on blood glucose after PIT (blue circles, vehicle; green circles, E2 in vivo; green triangles, E2 in vivo+in vitro). (d) Blood glucose AUC from (c). Values represent the mean ± SEM, *n* = 5–7/group. * *p* < 0.05. AS, after STZ; BS, before streptozotocin; V, vehicle. (PDF 31 kb)

ESM Figure 6 Effect of E2 treatment on macrophage infiltration after PIT. (a) Representative pictures showing macrophage infiltration determined by F4/80 antibody (green) in insulin cells (red) in the graft 3 day after PIT. The cell nuclei were stained with DAPI (blue). (b) Quantification of F4/80 positive cell numbers in the graft area from (a). Values represent the mean ± SEM, *n* = 4–5/group. V, vehicle. (PDF 105 kb)

ESM Figure 7 Effect of E2 on beta cell proliferation after PIT. (a) Representative pictures showing proliferating beta cells determined by Ki67 staining (green) in insulin^+^ cells (red) in the graft 3 days after PIT. The cell nuclei were stained with DAPI (blue). (**b**) Quantification of Ki67 positive beta cell numbers/insulin (+) area from (a). Values represent the mean ± SEM, *n* = 7/group. V, vehicle. (PDF 77 kb)

ESM Figure 8 ERα (E1644) expression in alpha cells. Western Blot indicating ERα (HC-20) production in different cell-lines. (PDF 30 kb)

## Abbreviations

17α-E2: 17α-Oestradiol
DPN: Diarylpropionitrile
E2: 17β-Oestradiol
ER: Oestrogen receptor
GPER: G protein-coupled oestrogen receptor
IEQ: Islet equivalents
JDRF: Juvenile Diabetes Research Foundation
PIT: Pancreatic islet transplantation
PPT: Propyl-pyrazole-triol
STZ: Streptozotocin
TBARS: Thiobarbituric acid reactive substances
