# Supplementary figures and images for: Oestrogens improve human pancreatic islet transplantation in a mouse model of insulin deficient diabetes
Source: Diabetologia. 2012 Nov 7;56(2):370–81. doi: 10.1007/s00125-012-2764-1 (PMC3536964; doi:10.1007/s00125-012-2764-1)

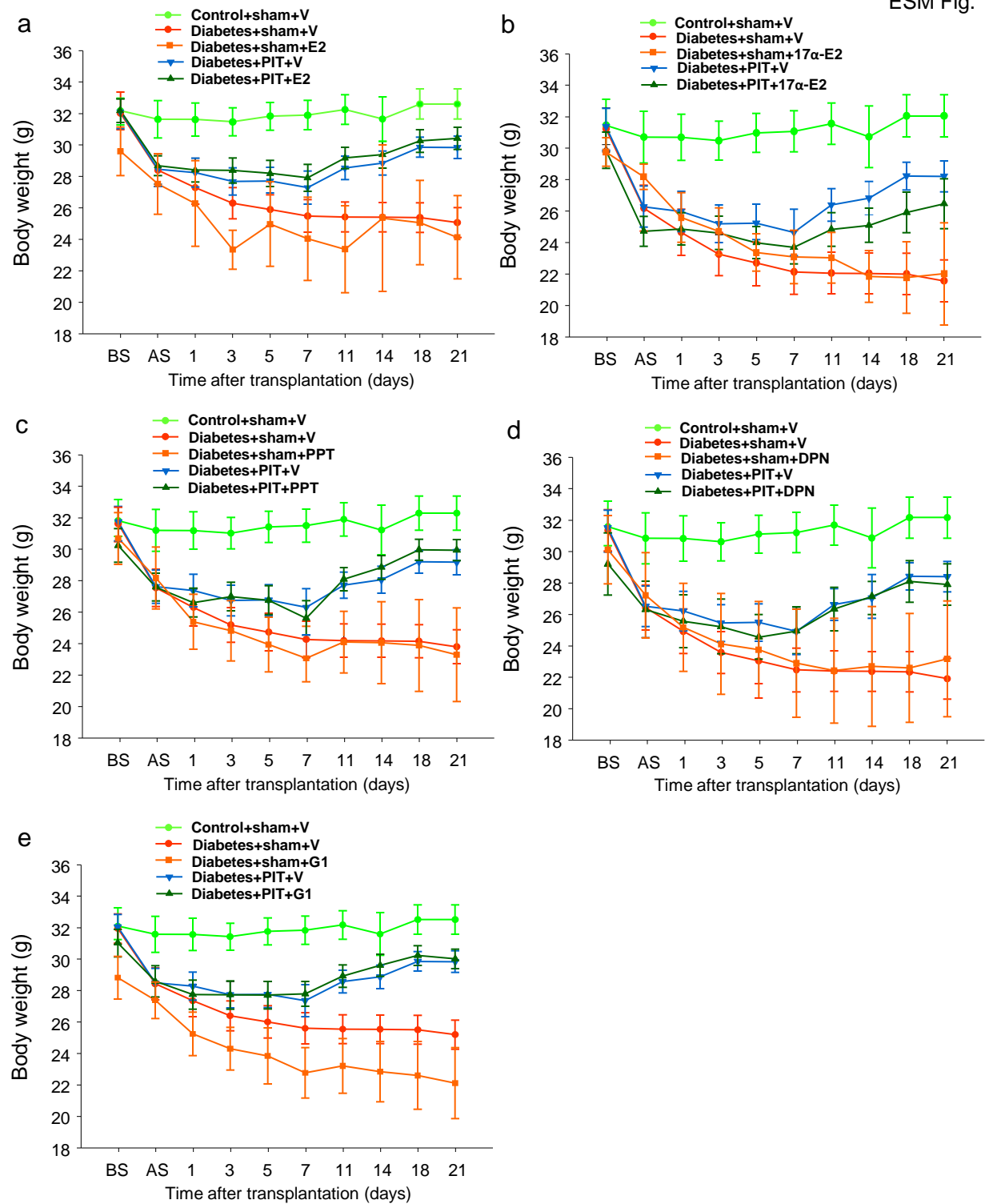

Supplement: Supplementary file 1 — Body weight after PIT. Effect of (a) E2, (b) 17α-E2, (c) PPT, (d) DPN and (e) G1 on body weight after PIT. Values represent the mean ± SEM. n = 4–18/group. AS, after STZ; BS, before streptozotocin; V, vehicle. (PDF 131 kb) [file 125_2012_2764_MOESM1_ESM.pdf]

a

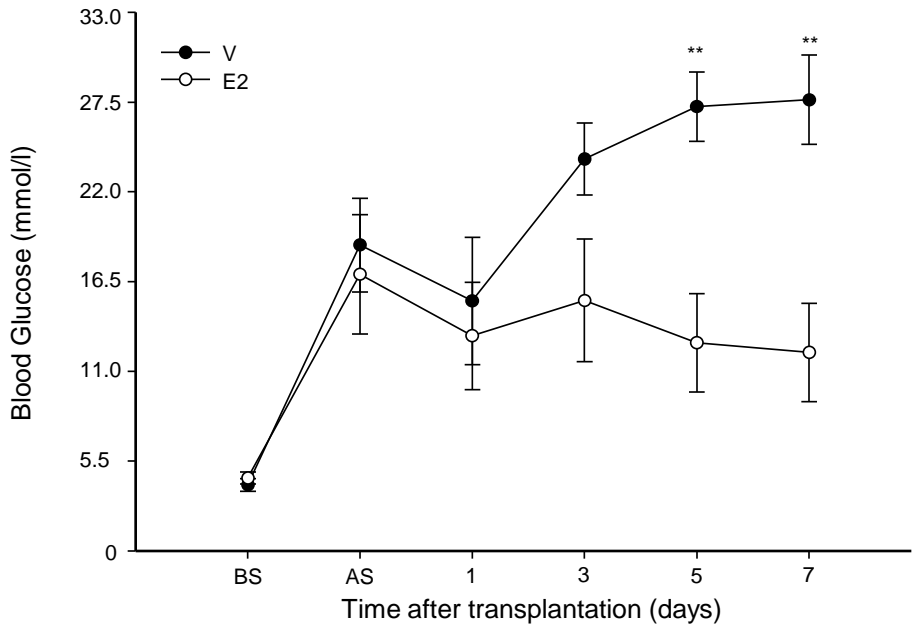

b

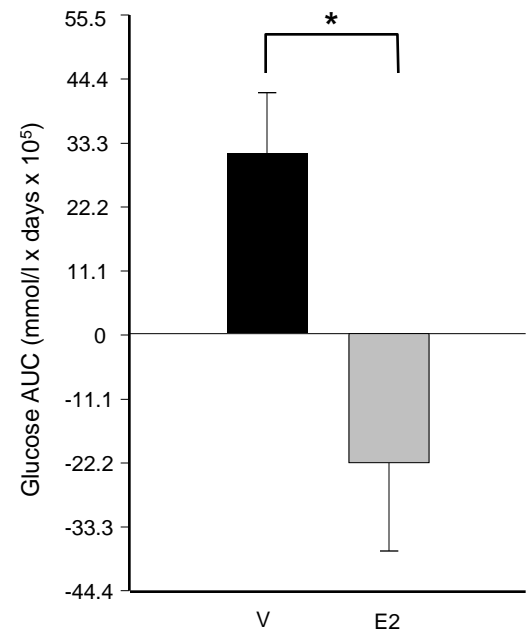

Supplement: Supplementary file 2 — Oestrogens ameliorate diabetes in female mice after PIT. (a) Effect of oestrogen on blood glucose levels in female mice (black circles, vehicle; white circles, E2). (b) Blood glucose area under the curve (AUC) was calculated from (a). Values represent the mean ± SEM, n = 4/group. *p < 0.05, **p < 0.01. AS, after STZ; BS, before streptozotocin; V, vehicle. (PDF 12 kb) [file 125_2012_2764_MOESM2_ESM.pdf]

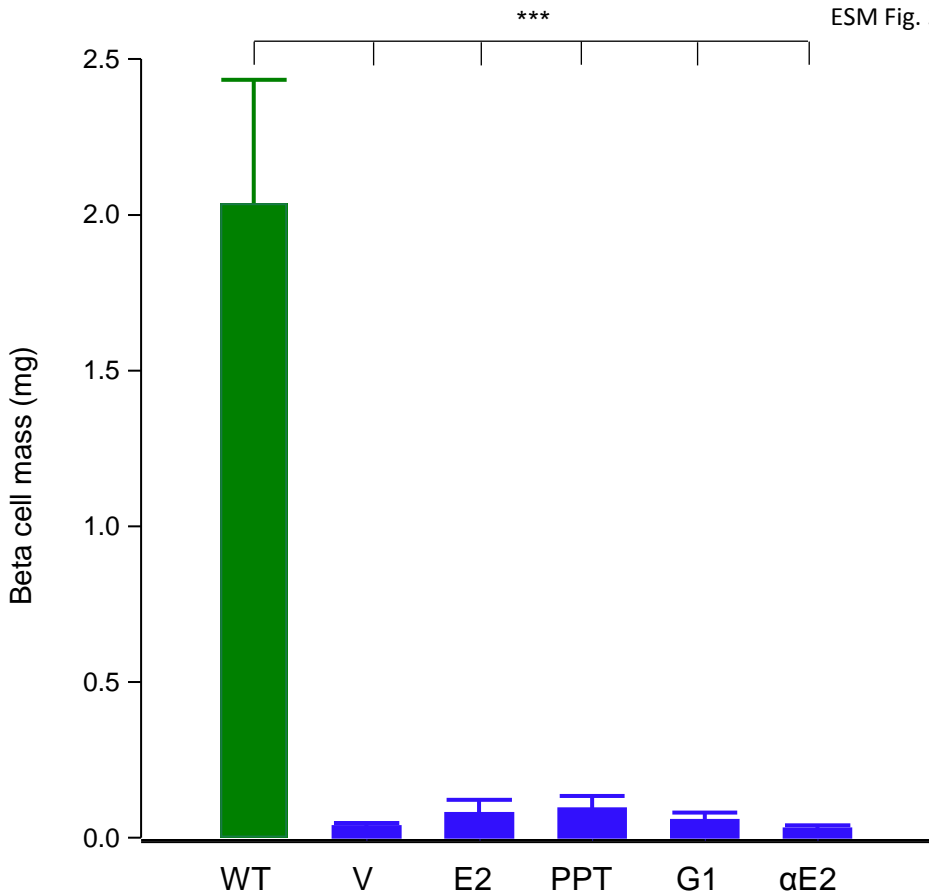

Supplement: Supplementary file 3 — Effect of oestrogens on pancreatic beta cell mass. Pancreatic beta cell mass was measured in sham operated control mice and PIT recipient mice treated with E2, 17α-E2, PPT, DPN, and G1. Values represent the mean ± SEM, n = 4–6/group. *** p < 0.001. V, vehicle. (PDF 70 kb) [file 125_2012_2764_MOESM3_ESM.pdf]

a

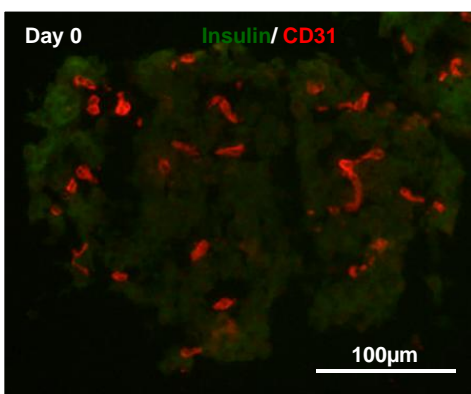

b

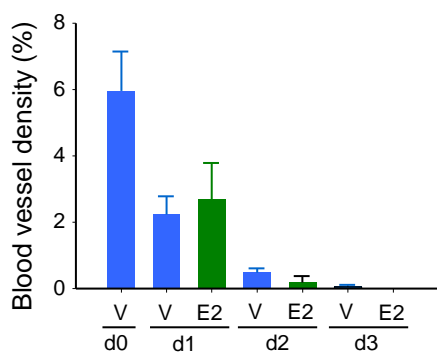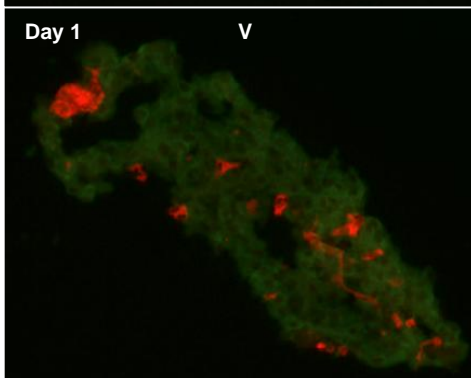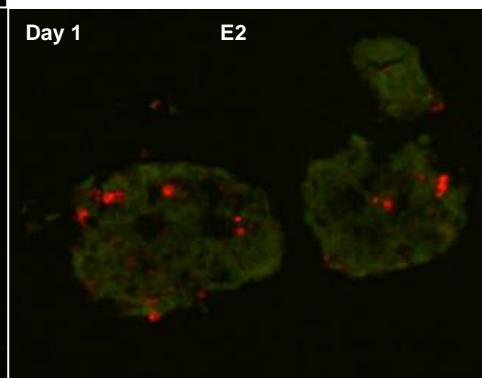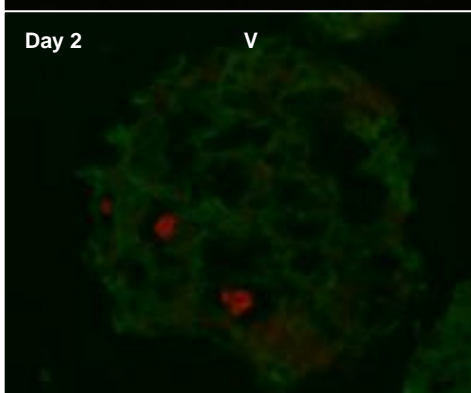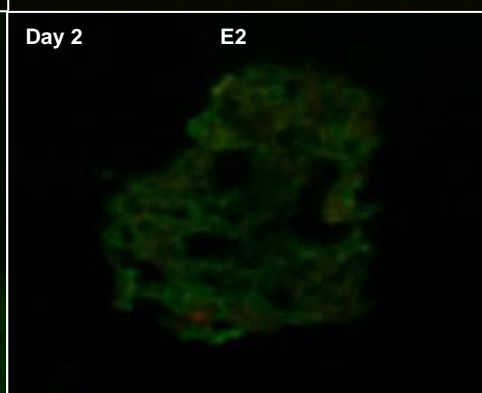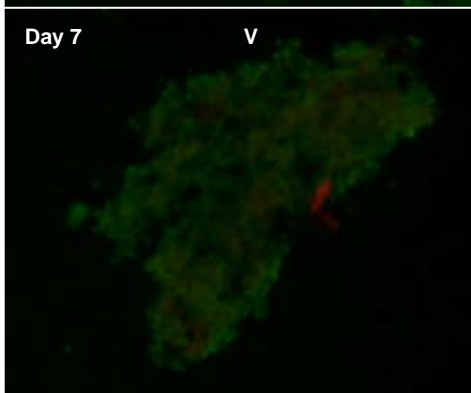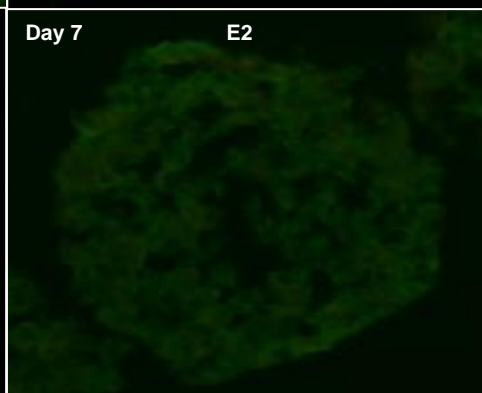

Supplement: Supplementary file 4 — Effect of in vitro E2 treatment on the retention of intra-islet endothelial cell population. (a) Representative sections showing immunofluorescence staining for mouse CD31+ (red) cells in cultured mouse islets (green). (b) Quantification of endothelial cell population in islets. Values represent the mean ± SEM, n = 3/group. V, vehicle. (PDF 50 kb) [file 125_2012_2764_MOESM4_ESM.pdf]

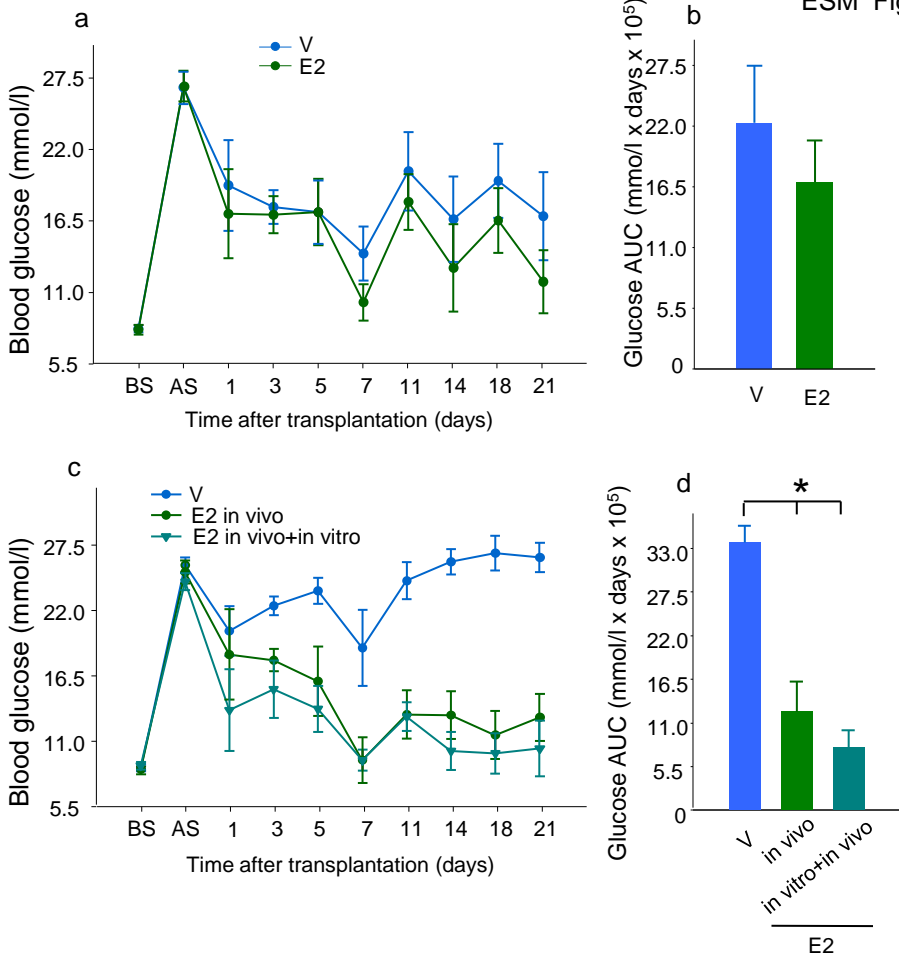

Supplement: Supplementary file 5 — Effect of in vitro E2 treatment on the PIT outcome. (a) Effect of in vitro E2 treatment alone (10−8 M) on blood glucose after PIT (blue circle, vehicle; green circle, E2). (b) Blood glucose area under the curve (AUC) from (a). (c) Comparison of dual in vitro and in vivo E2 treatment with in vivo E2 treatment alone on blood glucose after PIT (blue circles, vehicle; green circles, E2 in vivo; green triangles, E2 in vivo+in vitro). (d) Blood glucose AUC from (c). Values represent the mean ± SEM, n = 5–7/group. * p < 0.05. AS, after STZ; BS, before streptozotocin; V, vehicle. (PDF 31 kb) [file 125_2012_2764_MOESM5_ESM.pdf]

a

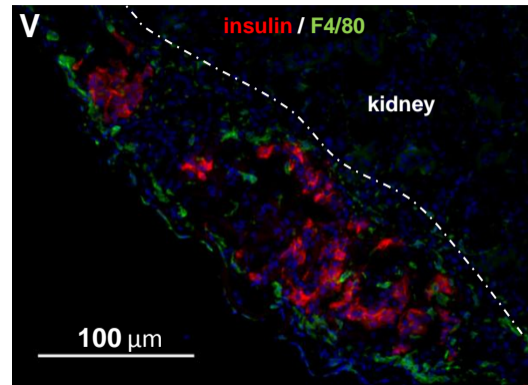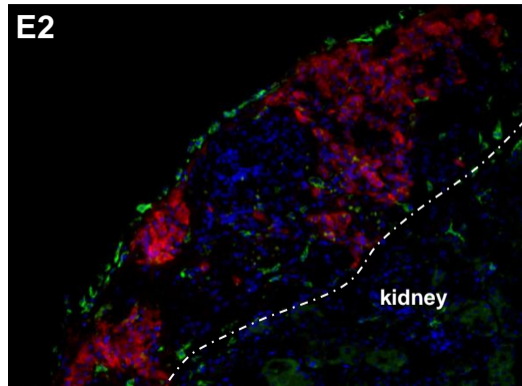

b

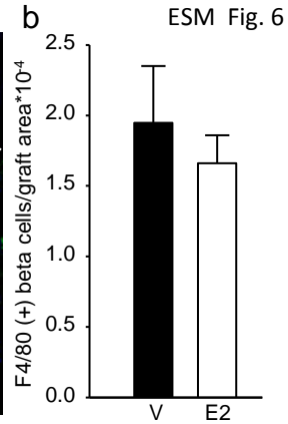

Supplement: Supplementary file 6 — Effect of E2 treatment on macrophage infiltration after PIT. (a) Representative pictures showing macrophage infiltration determined by F4/80 antibody (green) in insulin cells (red) in the graft 3 day after PIT. The cell nuclei were stained with DAPI (blue). (b) Quantification of F4/80 positive cell numbers in the graft area from (a). Values represent the mean ± SEM, n = 4–5/group. V, vehicle. (PDF 105 kb) [file 125_2012_2764_MOESM6_ESM.pdf]

a

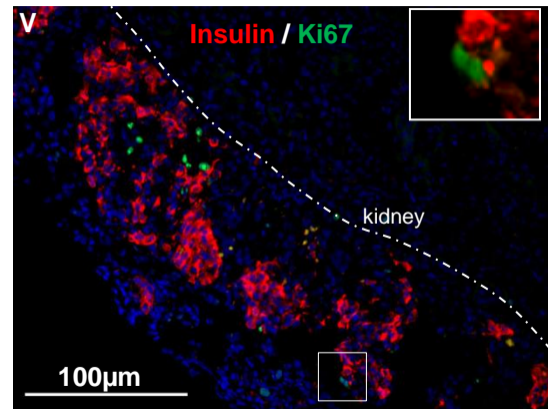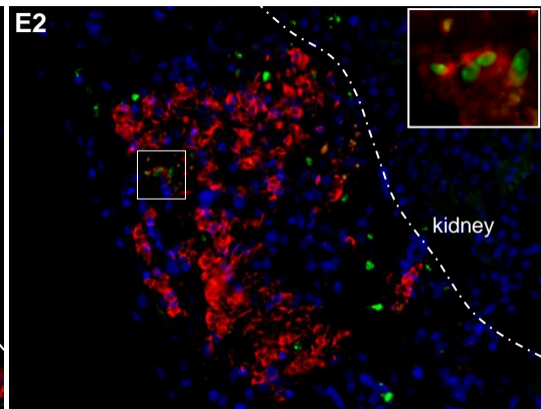

b

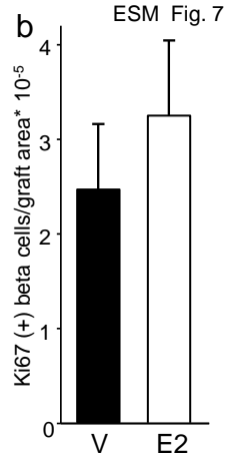

Supplement: Supplementary file 7 — Effect of E2 on beta cell proliferation after PIT. (a) Representative pictures showing proliferating beta cells determined by Ki67 staining (green) in insulin+ cells (red) in the graft 3 days after PIT. The cell nuclei were stained with DAPI (blue). (b) Quantification of Ki67 positive beta cell numbers/insulin (+) area from (a). Values represent the mean ± SEM, n = 7/group. V, vehicle. (PDF 77 kb) [file 125_2012_2764_MOESM7_ESM.pdf]

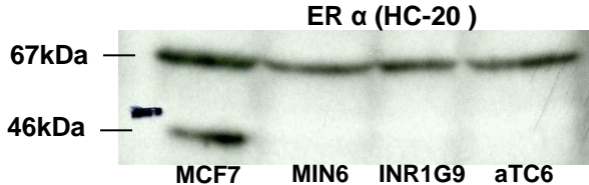

Supplement: Supplementary file 8 — ERα (E1644) expression in alpha cells. Western Blot indicating ERα (HC-20) production in different cell-lines. (PDF 30 kb) [file 125_2012_2764_MOESM8_ESM.pdf]
